# An App-Based Behavioral Support Intervention Promoting Physical Activity (APPROACH) in Patients Diagnosed With Breast, Prostate, or Colorectal Cancer: Protocol for a Randomized Controlled Trial

**DOI:** 10.2196/77096

**Published:** 2026-01-13

**Authors:** Fiona Kennedy, Sue L Smith, Rebecca J Beeken, Caroline Buck, Leanne Shearsmith, Hannah Truscott, Nicholas Counsell, Chloe Thomas, Anna Roberts, Diana M Greenfield, Henry WW Potts, Nicholas Latimer, Ed Lowther, Lee Smith, Rosie Stevens, Amy Creaser, Jacqui Gath, Lynda Wyld, Abigail Fisher, Phillippa Lally

**Affiliations:** 1 Leeds Institute of Health Sciences University of Leeds Leeds United Kingdom; 2 Department of Behavioural Science and Health University College London London United Kingdom; 3 Cancer Research UK & UCL Cancer Trials Centre, Cancer Institute University College London London United Kingdom; 4 Sheffield Centre for Health and Related Research School of Medicine and Population Health University of Sheffield Sheffield United Kingdom; 5 Sheffield Teaching Hospitals NHS Foundation Trust Sheffield United Kingdom; 6 Institute of Health Informatics University College London London United Kingdom; 7 UCL Advanced Research Computing Centre University College London London United Kingdom; 8 The Centre for Health, Performance and Wellbeing Anglia Ruskin University Cambridge United Kingdom; 9 Independent Cancer Patients' Voice (ICPV) London United Kingdom; 10 Department of Oncology and Metabolism University of Sheffield Sheffield United Kingdom; 11 University of Surrey Guildford United Kingdom

**Keywords:** app, cancer, cost-effectiveness, habit-based behavioral support, physical activity, quality of life, randomized controlled trial

## Abstract

**Background:**

Strong evidence highlights that sufficient physical activity (PA) has multiple benefits for people living with and beyond cancer. However, many are not meeting PA recommendations. APPROACH is a trial of a theory-driven, app-based behavioral support intervention to promote brisk walking after breast, prostate, or colorectal cancer.

**Objective:**

The aim of this trial is to evaluate the efficacy and cost-effectiveness of the intervention.

**Methods:**

APPROACH is a multicenter, phase III, 2-armed, individually randomized controlled trial (N=472). We will recruit patients with localized breast, prostate, or colorectal cancer from hospitals in Yorkshire and surrounding areas in the North of England, United Kingdom, and randomize them 1:1 between the intervention and control arm (usual care). The intervention consists of an app designed for the general population to encourage brisk walking (NHS Active 10), supplemented with habit-based behavioral support, including 2 brief telephone or video calls, a leaflet, website, and walking planners. The primary endpoint is the difference between trial arms in the changes from baseline in activPAL-assessed average minutes of brisk walking (≥100 steps per minute) after 3 months.

Demographic and medical characteristics will be collected through self-report and hospital records. Secondary outcomes (assessed at 0, 3, and 6 months) will be the other activPAL-assessed outcomes (brisk walking at 6 months, total steps, light PA, standing time, and sitting times, weekly metabolic equivalent of task), self-reported PA, and self-reported BMI and waist circumference. Patient-reported outcome measures of quality of life, fatigue, sleep, anxiety, depression, self-efficacy, habit strength for walking, and social support will also be collected. Interviews will explore experiences of receiving the intervention. We will use health economic modeling to estimate the cost-effectiveness of the intervention over a lifetime horizon.

**Results:**

The study was funded in June 2019. Trial recruitment commenced in November 2023 and is planned to be completed in 2025. As of December 2025, a total of 473 participants have been randomized. The publication of the main results is expected in autumn 2027 after all follow-up data collection and analysis are complete.

**Conclusions:**

Overall findings will determine the clinical and cost-effectiveness of the intervention for patients diagnosed with breast, prostate, or colorectal cancer. If successful, APPROACH provides a potential model of supportive care to increase PA among people living with and beyond cancer.

**Trial Registration:**

ISRCTN Registry ISRCTN14149329; https://www.isrctn.com/ISRCTN14149329

**International Registered Report Identifier (IRRID):**

DERR1-10.2196/77096

## Introduction

The global burden of cancer has more than doubled in the last 20 years [1,2]. Due to advancements in early detection and treatments [3], the number of people surviving cancer is increasing, with 5-year survival rates for some cancers such as breast cancer as high as 86% [4]. As survival rates continue to improve, there is a need to support the ever-growing population of people diagnosed with cancer, enabling them to live well and in good health [5].

The safety and feasibility of physical activity (PA) for oncology patients have been demonstrated by hundreds of trials. Meta-analyses exploring these have shown that PA is safe, feasible, and has beneficial effects on both physical and psychological functioning [6,7]. Benefits include reduced fatigue and pain, improvement in psychological and physical well-being [6,8], improved quality of life [9-13], and evidence of reduced risk of cancer-specific and all-cause mortality [14-19]. Breast, prostate, and colorectal cancer comprise 3 of the most commonly diagnosed cancers in the United Kingdom [20], and the evidence is strongest among these cancer types for the positive effects of PA after a cancer diagnosis [21-23].

Recognizing this evidence, the World Cancer Research Fund published recommendations for people diagnosed with cancer to engage in at least 150 minutes of moderate-to-vigorous physical activity (MVPA) per week, in line with those for the general population [24]. This report also highlighted that PA is not only exercise per se but includes everyday activities such as walking. More recently, these recommendations have been expanded to include efforts to reduce sedentary time. New guidance to “move more, sit less” encompasses the recognized benefits of an overall more active movement profile for people after a cancer diagnosis [25,26]. However, it is estimated that only around 30% of people diagnosed with cancer meet the recommended amounts of PA [27,28], and therefore there is a need to develop interventions that can increase PA levels in this population.

In the United Kingdom, a recommendation for PA advice for people diagnosed with cancer is included within national documents such as the Independent Cancer Taskforce [29], the 2019 National Health Service (NHS) Long Term Plan (albeit health and well-being focused) [5], and cancer charity reports [30]. Despite this and a documented desire among patients to receive advice [31,32], many people diagnosed with cancer do not recall receiving PA advice [33-35]. In one large study, less than a third of 15,254 people diagnosed with colorectal cancer recalled receiving any PA advice as part of their care [34]. In a survey undertaken with 242 people diagnosed with cancer in the United Kingdom, around half recalled receiving PA advice, but only 30% received guidance on the type of PA, and most (85%) were not referred to further information or an exercise specialist [36]. Reasons for this lack of PA advice in clinical practice include health care professionals not having the time and resources to provide advice, insufficient time in appointments, lack of knowledge of what to recommend, and not feeling they are the “right person” to deliver this advice [31,35,37]. These findings demonstrate the need for low-cost, scalable interventions that can be integrated into care and are accessible to a large number of patients.

Digital interventions offer the potential to reach wide populations, with smartphone apps becoming increasingly popular for harnessing behavior change. While a meta-analysis of 15 studies found that digital interventions could increase participation in MVPA by approximately 40 minutes per week in people diagnosed with cancer, only 2 of the included studies included delivery of the intervention by apps [38]. Apps can be used to monitor behaviors, track progress toward goals, and have been used to deliver “in the moment” behavior change techniques (BCTs), such as push notifications to remind people to complete activity [39]. Systematic reviews have also demonstrated that smartphone interventions offer potential to increase PA in people diagnosed with cancer and acknowledged the importance of cost-effectiveness when developing apps for intervention use, particularly focusing on the need to establish long-term changes in behavior that will be maintained [40,41].

To facilitate the maintenance of behaviors, habit theory posits that a habitual behavior is generated through a process where a context-response association is learned through repetition [42,43]. Theory suggests that contextual cues can then trigger the automatic execution of this behavior, even where motivation to perform the behavior in the moment is low [42,43]. Applying this theory in health behavior interventions could help encourage behavior maintenance beyond the intervention period, increasing the chances of long-term enactment of the health-promoting behavior [44,45].

This evidence suggests that an app-based intervention augmented with habit theory and personal contact has the potential to promote PA in people diagnosed with cancer. To explore this, the research group conducted qualitative user experience research with 31 people diagnosed with breast, prostate, or colorectal cancer who used 4 PA apps to identify their preferences [46]. No apps specifically designed to increase PA among people diagnosed with cancer were identified at the time, but the apps included were selected to represent different delivery formats (eg, tracking versus video instruction) and types of PA (eg, walking versus strength training), and were reviewed by a specialist oncology physiotherapist to check their safety for people with cancer [46]. In line with previous research [47], brisk walking was identified as the most achievable form of MVPA for people with cancer [46]. Participants also reported a preference for an app-based intervention that could be tailored to their ability and placed importance on the app being recommended by their health care professional, as well as being “badged” by or linked to the NHS [46]. The publicly available NHS Active 10 app [48] was chosen as it was the app that best captured all of these features, as well as including many of the key BCTs shown to be associated with successful PA change, including the focus on walking, goal setting, and self-monitoring [44,49-51]. Selecting an existing publicly available NHS-hosted app was a more cost-effective option with no development and maintenance costs, and it made it more likely that the app would be sustained and exist throughout our pilot, efficacy trial, and beyond. Qualitative research by our group with oncology clinical nurse specialists demonstrated that they would support an app-based intervention within the care pathway and would be happy to recommend this to patients, provided the app had demonstrated efficacy and was from a reputable source [52], the latter of which was fulfilled by using the NHS-developed and maintained Active 10 app, meaning implementation would be more likely.

This randomized controlled trial was informed by a pilot study [44] that demonstrated the feasibility and acceptability of our trial procedures and intervention content in 90 patients recruited from a single hospital in the United Kingdom. Small changes that were made to the intervention following the pilot are detailed in the “Intervention” section below [53]. The pilot study results also informed the sample size parameters for the trial’s primary endpoint. This study aims to investigate the efficacy of an app-based brisk walking intervention with behavioral support to increase PA in people diagnosed with breast, prostate, or colorectal cancer in the United Kingdom.

## Methods

### Primary Aim

The main objective is to increase brisk walking (activPAL-assessed average minutes ≥100 steps per minute [spm]) by using a theory-driven, app-based intervention that promotes brisk walking in adults living with breast, prostate, or colorectal cancer.

### Secondary Aims

The main secondary objectives are to compare the effect of the intervention with usual care on other activPAL-assessed outcomes (including average total steps, light PA, standing time and sitting time, weekly metabolic equivalent of task [MET], self-reported BMI and waist circumference, self-reported PA, habit strength for walking, PA self-efficacy, health status, quality of life outcomes, and health care usage) at 3 and 6 months. The cost-effectiveness of the intervention compared with usual care will be assessed as a secondary outcome. Engagement and satisfaction with the intervention will also be explored.

### Design

**Figure 1:**
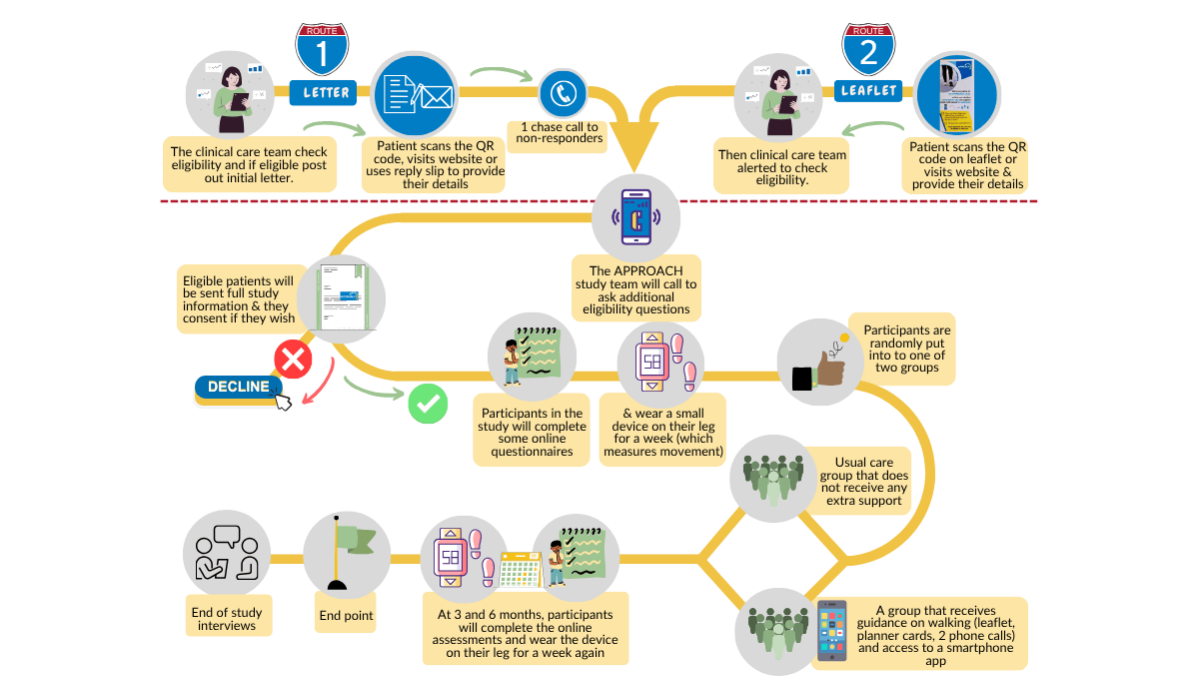
Flow chart of patient recruitment and involvement in study.

APPROACH is a multicenter, phase III, individually randomized controlled trial whereby people with a diagnosis of breast, prostate, or colorectal cancer are recruited and randomly allocated (1:1) between the intervention and control (usual care) arms. Study assessments are collected remotely at baseline, 3 months (12-18 weeks), and 6 months (24-30 weeks) after randomization. Figure 1 illustrates the flow of participants through the study. The trial has been designed following the CONSORT (Consolidated Standards of Reporting Trials) statement [54] and has been registered on the ISRCTN Registry [55]. The trial has been conducted in accordance with this trial registry record with no major deviations, only minor changes to trial documents, an extension to the recruitment timescale, and the addition of new recruitment hospital locations to boost recruitment. The reporting of this protocol follows the SPIRIT (Standard Protocol Items: Recommendations for Interventional Trials) guidelines (Multimedia Appendix 1 contains a completed SPIRIT checklist) [56]. A schedule of enrollment, interventions, and assessments based on the SPIRIT guidelines is shown in Table 1.

**Table 1:** SPIRIT (Standard Protocol Items: Recommendations for Interventional Trials) schedule of enrollment, interventions, and assessments.

Assessment and activity			Enrollment		Allocation		Post allocation				
			Any time during recruitment window				Weeks 1-12 (intervention)	3-month (12-18 weeks)		6-month (24-30 weeks)	Post study (30+ weeks)
**Enrollment**											
	Screening eligibility	✔									
	Eligibility confirmation	✔									
	Informed consent	✔									
	Allocation			✔							
**Intervention**											
	Intervention group					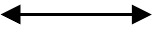					
	Usual care control group					✔					
**Assessments**											
	Demographics and diagnosis	✔									
	Individual differences (preference for routine and chronotype)	✔									
	Self-reported physical activity (GLTEQ^a^)	✔						✔	✔		
	Health-related quality of life (EQ-5D-5L)	✔						✔	✔		
	Cancer-specific quality of life (FACT-G^b^)	✔						✔	✔		
	Fatigue (FACIT-F^c^)	✔						✔	✔		
	Sleep (PSQI^d^)	✔						✔	✔		
	Anxiety (GAD-7^e^)	✔						✔	✔		
	Depression (PHQ-9^f^)	✔						✔	✔		
	Physical activity self-efficacy (PAAI^g^)	✔						✔	✔		
	Self-efficacy for self-management of cancer (CS-SES^h^)	✔						✔	✔		
	Habit strength for walking (SRBAI^i^)	✔						✔	✔		
	Social support (F-Soz-U K-6^j^)	✔						✔	✔		
	Health and social care service use (CSRI^k^)	✔						✔	✔		
	Height, weight, and waist circumference (optional)	✔						✔ (not including height)	✔ (not including height)		
	Accelerometry (activPAL) (3-7 days)	✔						✔	✔		
	Poststudy qualitative interviews										✔ (intervention group only)
	Online Intervention feedback questionnaire							✔ (intervention group only)	✔ (intervention group only)		

^a^GLTEQ: Godin Leisure Time Exercise Questionnaire.

^b^FACT-G: Functional Assessment of Cancer Therapy-General.

^c^FACIT-F: Functional Assessment of Chronic Illness Therapy-Fatigue.

^d^PSQI: Pittsburgh Sleep Quality Index.

^e^GAD-7: Generalized Anxiety Disorder-7.

^f^PHQ-9: Patient Health Questionnaire-9.

^g^PAAI: Physical Activity Appraisal Inventory.

^h^CS-SES: Cancer Survivorship Self-Efficacy Scale.

^i^SRBAI: Self-Report Behavioral Automaticity Index.

^j^F-Soz-U K-6: 6-item brief form of the Perceived Social Support Questionnaire.

^k^CSRI: Client Service Receipt Inventory.

### Eligibility Criteria

Table 2 outlines the inclusion and exclusion criteria across the eligibility screening phases.

**Table 2:** Eligibility criteria for each screening phase.

Screening phase	Inclusion criteria	Exclusion criteria
Phase 1: medical record (NHS^a^ site)	Confirmed diagnosis of breast, prostate, or colorectal cancer within the past 2 years and registered as a patient at one of the recruiting hospitals Aged ≥16 years	Diagnosed with a cognitive impairment (eg, dementia) Cognitive or physical impairment that prevents participation in brisk walking^b^ Metastatic disease Incarcerated
Phase 2: participant screening by telephone (APPROACH team)	Owns a smartphone using either Android or iOS operating systems Willing and able to provide informed consent Has access to a computer or tablet and an email address, and is willing to complete online questionnaires Willing to wear an accelerometer for 1 week at each assessment point	More than 2.5 years postdiagnosis at consent^c^ Unable to understand spoken or written English Eastern Cooperative Oncology Group performance status of ≥3 (limited self-care; confined to bed or chair for ≥50% of waking hours) Cognitive or physical impairment that prevents participation in walking^b^ Scheduled to undergo surgery to remove cancer within the next 5 months at the time of screening Fewer than 6 weeks after surgery for cancer removal at baseline assessment Report ≥150 minutes of at least moderate-to-vigorous physical activity per week Report previous or current use of the intervention app Currently participating in another health behavior change study (eg, diet, physical activity, alcohol intake, and weight management) Lives with someone already participating in the trial Participated in the pilot study (only applies at one site)

^a^NHS: National Health Service.

^b^This criterion is screened twice: first in the medical records and again with participants.

^c^The 6-month gap between medical-record screening (<2 years postdiagnosis) and direct participant screening (2.5 years postdiagnosis) recognizes the potential time between screening and consent.

### Recruitment and Setting

Recruitment takes place across multiple hospitals in Yorkshire (our funding stipulates that the majority of recruitment is from the Yorkshire area) and surrounding areas in the North of England, United Kingdom (sites are listed on the ISRCTN trial record [55]). At each site, the clinical care team identifies patients who have been diagnosed with breast, prostate, or colorectal cancer within the last 2 years and examines their medical records for the phase 1 inclusion criteria (Table 2). NHS staff mail or email eligible patients a letter about the study asking them to indicate (by online form, paper reply slip, or telephone) whether the APPROACH team can contact them. If site capacity allows, after at least 2 weeks, nonresponders are telephoned or emailed by hospital staff. Study leaflets and poster advertisements to be distributed in hospital waiting rooms and clinic areas are also available, enabling patients to self-refer (self-referral triggers a check of phase 1 eligibility by the NHS team).

If a patient expresses interest and agrees to a call, the APPROACH team telephones to conduct phase 2 eligibility screening (Table 2) and provide the participant information sheet by email if eligible. The participant information sheet was redesigned following the pilot study to ensure the language was accessible, the format easy to read (including a visual infographic to describe trial activities), and the images culturally sensitive and relevant. These materials were also reviewed and approved by our patient and public involvement members. The email links to a REDCap (Research Electronic Data Capture) online website [57,58] (electronic survey software hosted within the encrypted Data Safe Haven platform at University College London [UCL]) where the full participant information sheet is displayed and the online consent form provided (Multimedia Appendix 2). Patients can request a paper information sheet if desired. Consented participants complete the baseline assessments comprising an activPAL (activPAL4micro accelerometer; PAL Technologies Ltd), an online questionnaire, and optional weight, height, and waist circumference measurements (provision of weighing scales and tape measure). Participants can also opt into allowing access to and sharing of their NHS number with Hospital Episode Statistics (HES) and the National Disease Registration Service (NDRS) registries to enable exploration of long-term (10-year) health outcomes.

### Sample Size

The APPROACH pilot study data [53] were used to inform this power calculation. The target sample size is 472 participants randomized 1:1 to detect a difference between trial arms in the change from baseline in the daily average of 6 minutes per day (ie, 42 minutes per week) of activity at 100 spm or higher. This assumes 90% power, a 2-sided 5% significance level, an SD of 12 minutes per day in the control arm, and a variance ratio of 1:4 (control:intervention). Low attrition (3%) was observed in the pilot, but with multiple recruiting sites and longer follow-up, a 10% attrition rate has been allowed to ensure the required 212 participants per arm is achieved.

### Randomization

Post baseline data collection, participants are individually randomized using minimization with a 1:1 allocation ratio by the central trial team at UCL (a researcher not involved with recruitment or data collection) using code developed by a UCL senior research data scientist, based on the paper by O’Callaghan [59], which is available on GitHub [60]. Stratification factors are cancer type (breast, prostate, colorectal) and hospital site. The first participant is randomly allocated; thereafter, subsequent participants are assigned based on the arm with the lowest imbalance score while factoring in a 20% random element. Randomization constitutes full enrollment in the trial.

### Intervention

#### Overview

The intervention consists of the app (NHS Active 10) plus supplementary brief written and telephone behavioral support [44,53] (Multimedia Appendix 3 provides a Template for Intervention Description and Replication [TIDieR] checklist). The intervention has been designed and coded according to the Behavior Change Taxonomy Ontology reported by Michie et al [61] (Table 3 shows the intervention component mapping carried out by PL, FK, CB, SLS, and AF) [62,63].

**Table 3:** Intervention components and mapping to the Behavior Change Taxonomy Ontology (BCTO) items.

Intervention components and BCTO items				BCTO item keys^a^
**Active 10 App**				
	Introducing the app into participants’ environment			007156
	The App is hosted by NHS^b^ Better Health			007075
	Introduction: “Brisk walking is…” “Every minute counts” “Aim for 10 min or more a day”			007058
	I am doing Active 10 because…			007005
	Set your targets (1, 2, or 3 Active 10s a day)			007300
	If regularly achieve 3 Active 10s a day can set a target of 4 or 5			007100
	Walking tracker (minutes of walking and minutes of brisk walking)			007023
	Rewards			007253, 007254, 007209, 007211
	**Optional links and functions, including a variety of “tips,” which participants may or may not access**			
		Links to useful websites (eg, NHS, Strava)	N/A^c^	
		Link to a discussion group for the app	007029	
		Articles on physical and mental health benefits of physical activity, disabilities, how much physical activity to do, other ways to improve mental health (eg, meditation)	007063, 007065	
		Ability to set reminders	007080	
		Pace checker	007017	
		Tip: How fast is a “brisk” walk	007042	
		Tip: set a reminder	007080	
		Tip: keep track (use app to see how you are doing)	007023	
		Tip: schedule a time for a walk	007010	
		Tip: walk with a friend	007029	
		Tip: prepare (get shoes ready)	007168	
		Tip: Listen to music or podcast	007061	
		Tip: reward yourself	007187	
**Leaflet and accompanying letter from clinical team**				
	Clinical team recommendation to read and use the information provided and to download Active 10			007074, 007075, 007077
	Letter and leaflet informing that Active 10 was developed by NHS			007075
	Branding: Yorkshire Cancer Research, UCL^d^, University of Leeds, University of Sheffield, Anglia Ruskin University, and Sheffield Teaching Hospitals.			007075
	Physical activity improves side effects of cancer treatment, recovery and risk of recurrence, mood and confidence. Physical activity reduces risks of other health problems.			007183, 007181
	Quotes from patients with cancer: used physical activity to cope with fatigue, chose walking to try to meet guidelines			007074, 007183, 007181
	People who have or have had cancer recommended to try to meet the same physical activity guidelines as other adults. Brisk walking 2-3 times every day will meet the activity guidelines (150 minutes); the more, the better			007075, 007076
	Recommends brisk walking. This should make you breathe a bit faster…			007058
	Recommends start small then build up			007100
	Information on downloading Active 10			007058
	Recommends planning			007008
	Recommends walking at the same time or in the same situation			007094, 007096
	Recommends tracking behavior using the walking planners and Active 10			007076
	Recommends using the website for more information			N/A
**Walking planner**				
	Adding planner to people’s environment			007164
	Promotes habit formation			007094, 007096
	Promotes adapting plans as needed			007299
	If your days are different, try to develop a few different habits...			007010
	How many Active 10s are you aiming for			007003
	Plan: when and how much			007010
	Did you complete your plans?			007024
	How did you feel after you walked briskly?			007066, 007025
	Reminder not to worry if you miss a day and to adjust plans as required (reduce if finding it hard and increase if meeting goals and feel able).			007299
**Website**				
	Website and video branded from Yorkshire Cancer Research			007075
	Links to resources about the health benefits of physical activity			007183, 007181
	Links to resources to support walking (general walking websites and local information for areas recruiting from)			007042
	Promote physical and mental health benefits of brisk walking			007183, 007181
	Video: (1) promotes the physical and mental health benefits of brisk walking; (2) states that participants report enjoying using the app, liking having control and doing something for their health, and liking monitoring progress; and (3) shows a participant describing how they built up walking over time, made it part of their routine, and reported less physical pain and feeling emotionally better			007175, 007068, 007120, 007072, 007076
**Intervention: phone call or video call (1)**				
	Introduce self as working with clinical team at the hospital			007075
	Ask participants how their cancer and treatment have impacted their lifestyle and activity levels			
	Discuss physical and mental health benefits of physical activity			007183, 007181
	Discuss motivations to increase activity			007069
	Discuss concerns about increasing activity			007069, 007008
	Help participant to work out ways to overcome concerns about brisk walking, provide information as appropriate			007008
	Discuss why recommending brisk walking, including cancer patients have recommended this			007074
	Describe brisk walking			007058, 007051
	Provide information on government guidelines (150 minutes of MVPA^e^) as well as WHO^f^ Every Move Counts.			007075
	Highlight building up over time			007100
	Discuss how confident they are and how they can increase their confidence			007008
	Suggest trying it to see if that increases their confidence			007054, 007137
	If needed tell them that it is possible for them to do this and others have been able to			007137, 007076
	Promote habit formation for initiating a walk			007094, 007096
	Promote habit formation for planning walks, looking at app or planner			007094, 007096
	Make an action plan (when, what, how long)			007010
	Promote self-reward during or after walking			007257, 007252
	Promote nonspecific self-reward during or after walking			007257, 007252
	Promote using the app to track activity			007026
	Promote specific cues			007080, 007081
	Promote reminders			007080, 007081
	Promote adjustment of goals if needed			007011
	Promote adjustment of plans if needed			007299
	Set a target number of Active 10s			007300, 007010
	Promote asking friends to support, by encouraging and helping to remember to walk			007030, 007031, 007032, 007033
	Promote using the walking planner to track behavior			007024, 007025
	Encourage participants to use information provided to overcome their concerns about exercising			007008
**Intervention: phone call or video call (2)**				
	Remind them of their target, and discuss if needs adapting			007011
	Ask how they are getting on with their target			007012
	Ask participants what is preventing them from walking and what would help them to start (if relevant)			007008
	Repeat any of the points from call 1 as appropriate			As relevant

^a^BCTO item key: 007003 set behavior goal behavior change technique (BCT); 007005 set outcome goal; 007008 goal strategizing BCT; 007010 action planning; 007011 review behavior goal BCT; 007012 attend to discrepancy between current behavior and goal BCT; 007017 monitoring BCT; 007023 provide feedback on behavior; 007024 self-monitor behavior BCT; 007025 self-monitor outcome of behavior BCT; 007026 provide biofeedback BCT; 007029 advise to seek support; 007030 advise to seek instrumental support BCT; 007031 advise to seek emotional support BCT; 007032 advise to seek informational support BCT; 007033 advise to seek appraisal support BCT; 007042 deliver informational support; 007051 agree on how to perform behavior BCT; 007054 conduct a behavioral experiment BCT; 007058 instruct how to perform behavior; 007061 plan inclusion of enjoyment; 007063 inform about health consequences; 007065 inform about emotional consequences; 007066 monitor emotional consequences BCT; 007068 increase salience of consequences BCT; 007069 consider pros and cons BCT; 007072 awareness of other people’s thoughts, feelings, and actions BCT; 007074 increase awareness of others approval BCT; 007075 present information from credible influence BCT; 007076 suggest to change behavior BCT; 007077 tell to change behavior BCT; 007080 prompt intended action BCT; 007081 cue BCT; 007094 practice behavior BCT; 007096 context-specific repetition of behavior BCT; 007100 set graded tasks BCT; 007120 vicarious reward BCT; 007137 persuade about personal capability BCT; 007156 add objects to the environment BCT; 007164 add objects to the indirectly experienced environment BCT; 007168 advise specific behavior BCT; 007175 increase salience of behavior BCT; 007181 inform about positive emotional consequences BCT; 007183 inform about positive health consequences BCT; 007187 promise consequence for behavior BCT; 007209 promise positive material consequence for behavior BCT; 007211 promise positive material consequence for approximating behavior BCT; 007252 provide positive consequence for behavior BCT; 007253 provide positive consequence for approximating behavior BCT; 007254 provide positive consequence for completion of behavioral sequence BCT; 007257 provide positive material consequence for behavior BCT; 007299 review behavior goal plan BCT; and 007300 set measurable behavior goal BCT.

^b^NHS: National Health Service.

^c^N/A: not applicable.

^d^UCL: University College London.

^e^MVPA: moderate-to-vigorous physical activity.

^f^WHO: World Health Organization.

Several BCTs have shown promise in systematic reviews and meta-analyses focused on increasing PA in inactive adults [49] and specifically after cancer, including adding an object to the environment, self-monitoring, goal setting [64], action planning, social support [65], and rewards [66]. This study focused on BCTs that were considered to have efficacy in this population and those practical to use in the context of an app-based intervention with brief behavioral support.

Intervention participants are mailed a pack containing a clinician endorsed letter, alongside printed materials (leaflet and walking planner cards) and an appointment time for a behavioral support video or telephone call. Since the trial is ongoing at the time of writing this protocol, intervention materials are not included, but the mapping exercise is provided (Table 3) and further detail is available upon request from the corresponding author.

#### Leaflet and Website

The leaflet covers topics including the benefits of PA during or after cancer treatment (eg, reduces side effects), introduces how walking and particularly brisk walking counts as moderate PA (encouraging individuals to gradually build up), and walking habits (eg, importance of repetition, planning walking into daily routines). The leaflet also introduces the NHS Active 10 app (including how to download it), and has a link to the study website (not used in the pilot as all information was included in the leaflet because the pilot was conducted in one location), which contains copies of all the study resources and also local area-specific resources relating to walking groups or places to walk, which were accumulated by doing internet searches of walking resources in these areas (eg, Get Doncaster Moving council initiative, 5k Your Way). The website used in the randomized controlled trial also includes a brief film of participants from the pilot [53] who used the app and increased their walking.

#### Walking Planner Cards

The walking planner cards are underpinned by BCT principles and habit theory that have been shown to increase PA, including a focus on helping participants set goals (eg, “How many daily Active 10s are you aiming for?”), plan their walks (action planning, eg, “On Monday I will walk:…”), and self-monitor (eg, “Tick when you complete each plan”). These cards were amended based on feedback from pilot participants [53], many of whom felt they were too restrictive. The core content remained (eg, prompting the time of day or situation), but the redesign enabled more flexibility for different plans on different days of the week.

#### App

The NHS Active 10 app is publicly available, developed by Public Health England [48], and currently maintained by the Department of Health and Social Care. This app was selected based on extensive development work [46,52,67], plus patient and public involvement (PPI) and pilot study results [53]. The app incorporates BCTs such as goal setting, self-monitoring behavior, and prompting practice of behaviors [68,69].

The app tracks all walking activity and provides a total for time spent walking and time spent brisk walking. Ten minutes of brisk walking is known as one “Active 10.” The app encourages users to initially build up to completing one Active 10. Users can set a goal to complete 1-3 Active 10s per day, with the aim of reaching 30 minutes, and if successful they can set up to 5 Active 10s. Each individual minute of brisk walking is recorded and contributes to the Active 10s (to fit with current UK Chief Medical Officers’ PA guidance that updated the previous recommendation that a minimum 10-minute bout of activity was required [70]). Moderate activity has been shown to provide greater health benefits than slower-paced walking [71]. The app uses the mobile phone’s inbuilt sensors to measure activity and record the total minutes of walking and, where relevant, brisk walking (100+ spm).

**Figure 2:**
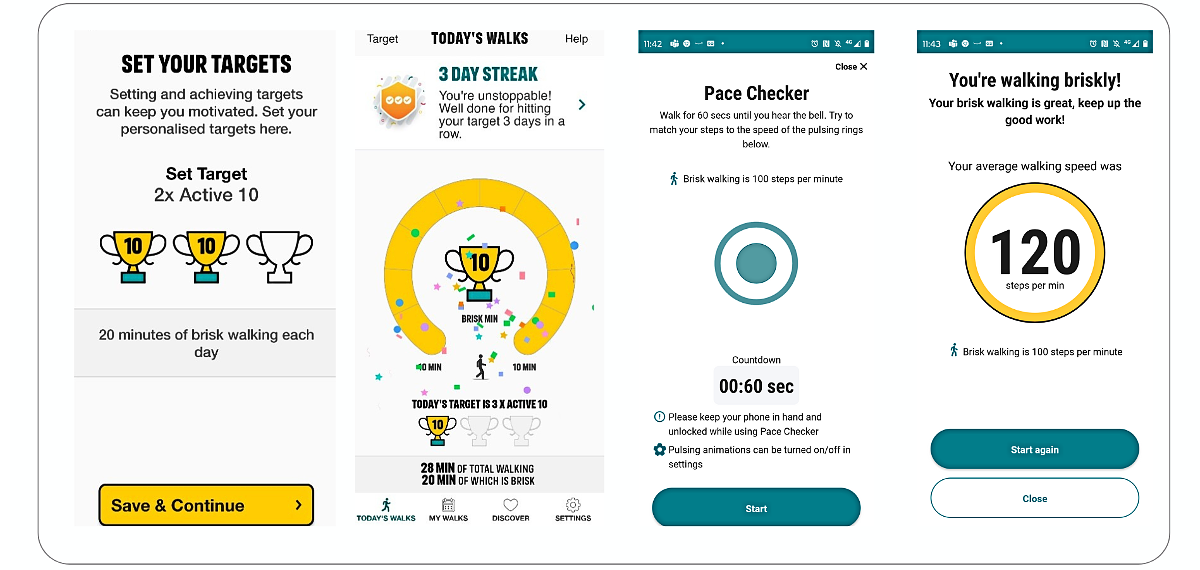
Screenshots of Active 10 app.

Since the pilot study was completed, a “pace checker” function has been added, enabling users to see timed pulses on the app (in line with 100 spm) while walking for a 1-minute period and, at the end of the 1 minute, shows the number of steps achieved and whether it was classed as brisk. Figure 2 illustrates some screenshots from the app, including the new feature.

#### Behavioral Support Calls

Two semiscripted behavioral support video or telephone calls are undertaken by a trained researcher around one month apart. The first call takes place as soon after randomization as possible and introduces the recommendations around PA for people diagnosed with cancer (eg, 150 minutes of MVPA), how brisk walking is a form of moderate PA, the evidence for benefits of meeting these guidelines, and the importance of increasing PA by any amount (eg, highlighting the Every Movement Counts World Health Organization campaign) [72]. The researcher also explains how to form walking habits, how the app works, and encourages use of the app to help set and monitor goals (eg, “What do you want to aim for? One, two or three active tens [so 10, 20 or 30 minutes of brisk walking a day]”). Participants are encouraged to make plans and establish habits for using the app, plan to increase their brisk walking if appropriate, and use the app to track their progress. The call also aims to explore barriers to PA and explore ways of addressing them drawn from evidence (eg, treatment-related fatigue). This call is intended to replicate the type of conversation that a health care professional (eg, clinical nurse specialist) could have with patients as part of routine care. A second call is arranged for around 4 weeks later to see how participants are progressing, troubleshoot (eg, their plans or the app), and remind and recap on any information as required. With the participant’s agreement, calls are recorded to allow fidelity checks.

### Control Group

Control group participants are notified of their group allocation by telephone or email, then receive “usual care” (no additional support beyond being informed in the information sheet that PA is beneficial for people after a diagnosis of cancer).

### Measures

#### Overview

For all assessments, participants are emailed a link to a questionnaire on REDCap [57,58] and mailed an activPAL. If participants consented to provide body measurements, they are also mailed a set of weighing scales (Seca 803 if they weigh less than 150 kg; Seca 813 if they weigh over 150 kg) and a tape measure (Seca 201) at baseline. Reminder calls are made if any questionnaires are not completed, and telephone support on completing the questionnaires is provided where necessary. To be included in analysis, 3-month data must be completed between 12-18 weeks postrandomization and 6-month data between 24-30 weeks postrandomization. Researchers involved in collecting follow-up assessment data are blinded to group allocation.

#### Sociodemographic Characteristics and Disease Characteristics

At baseline, age, sex, employment and education status, marital status, living arrangements, postcode, and ethnicity are self-reported. Participant’s postcodes will be used to determine socioeconomic position using the Index of Multiple Deprivation [73]. Participants are asked to self-report their cancer diagnosis and comorbid health conditions [74] at baseline, and clinical details will be verified from medical records (including diagnosis date and stage and treatment received).

#### Physical Activity

PA is measured objectively [75] using thigh-worn activPAL accelerometer (sent with detailed instructions and materials to apply the device to their thigh, plus a link to a video of a researcher demonstrating how to attach the device). Participants are asked to wear the activPAL for 7 days continuously at each timepoint (ie, baseline, 3 months, and 6 months). ActivPAL data will be used to calculate the primary endpoint. Participants will also complete a log sheet recording when the device was worn and their bedtimes and waketimes. ActivPAL devices have been used successfully in other studies, including those diagnosed with cancer [76], and were used in the pilot [44,53]. The primary endpoint is activPAL-assessed average minutes of brisk walking (≥100 spm) 3 months after randomization (controlling for baseline rates). ActivPAL data will also be used to assess brisk walking at 6 months, average total steps, minutes of light PA, standing time, sitting time, and weekly MET. Participants will also complete the Godin Leisure Time Exercise Questionnaire (GLTEQ) [77], which has demonstrated favorable validity and reliability against objective measures of PA and is widely used in oncology research [78]. This questionnaire was adapted in the pilot [44,53] to include a question about duration of activities to allow the calculation of minutes of MVPA [78].

#### Anthropometric Outcomes (Optional)

Height, weight (without outer clothing or shoes on), and waist circumference (at the umbilicus) are self-reported by participants into an online questionnaire. Previous studies have illustrated that self-reported weight and waist circumference are adequately reliable and accurate where objective measurement is not possible [79-81]. BMI will be calculated using the standard formula of weight (kg) ÷ height (m)^2^. Participants who provide these measures at baseline will keep the weighing scales and tape measure for the later follow-ups.

#### Psychosocial Variables

To assess the broader impact of the intervention, the psychosocial variables outlined in Table 4 are self-reported at each timepoint.

**Table 4:** Details of the psychosocial variables and measures included in the questionnaires.

Construct	Measure	Reliability and validity references
Health related quality of life (HRQoL)	EQ-5D-5L and visual analogue scale [82]	Reliable and valid among patients with cancer [83]
Cancer-specific quality of life	27-item Functional Assessment of Cancer Therapy-General (FACT-G) scale [84]	Good validity and reliability [84]
Fatigue	13-item fatigue subscale of the Functional Assessment of Chronic Illness Therapy-Fatigue (FACIT-F) [85]	A brief reliable and valid measure [85]
Sleep quality	18-item Pittsburgh Sleep Quality Index (PSQI), which assesses sleep quality and disturbances over a 1-month time interval [86]	Used extensively in clinical (including cancer) and nonclinical samples, with good reliability and validity [87]
Anxiety	7-item Generalized Anxiety Disorder-7 (GAD-7) Assessment [88]	Adequate diagnostic accuracy in patients with cancer [89]
Depression	9-item Patient Health Questionnaire (PHQ-9) [90]	Adequate diagnostic accuracy in patients with cancer [91]
Physical activity self-efficacy	13-item Physical Activity Appraisal Inventory (PAAI)	Reliability and validity in women with breast cancer [92]
Self-efficacy to self-manage cancer	11-item Cancer Survivorship Self-Efficacy Scale (CS-SES) [93]	Excellent reliability across mixed patients with cancer [93]
Habit strength for walking	4-item Self-Report Behavioral Automaticity Index (SRBAI) [94], which will be measured focusing on “going for a walk” and “walking briskly”	Good reliability and sensitivity to changes in habit [94]
Social support	6-item brief form of the Perceived Social Support Questionnaire (F-Soz-U K-6) [95]	Good reliability and validity across the lifespan [95]

To conduct exploratory analysis between the activPAL data and habit strength, investigating whether those who walk in the morning have higher habit strength than those who walk in the evening, participants will be asked 4 questions related to their chronotype (individual differences in sleep timing and in preferences for sleeping at given times of day). This will be measured using a subscale of the Morningness-Eveningness Questionnaire [96] used by Randler et al [97], with the English wording taken from Terman et al [98]. Additionally, 2 items (scored on a 5-point Likert scale) from the Creature of Habit scale [99] will be used to assess preference for routine (“I tend to like routine” and “I find comfort in regularity”).

#### Health and Social Care Service Use

Participants are asked to self-report their use of health and social care using items from the Client Service Receipt Inventory (CSRI), which has been validated against objective primary care records [100] and is recommended for usage of hospital and other community health services [101]. As part of the medical records data collection (undertaken by research staff at 6 months postrandomization), details about participant’s hospital-based treatment and care received (including the number of hospital contacts) during the 30-week trial period will be recorded.

#### App Use and Physical Activity Changes

No data collected by the app itself will be obtained by the research team [53] owing to the app data not being stored in a way that would allow linkage with our trial participants. Therefore, participants in both groups are asked to report their use of any PA apps during the study period, whether they have done anything to change their PA since starting the trial, and what prompted them. Additionally, intervention group participants complete brief feedback questions on the intervention content in the follow-up questionnaires, self-report their usage of the app, and complete the Digital Behavior Change Interventions Engagement Scale [102], which was used in the pilot study to capture app engagement [53]. A single item (“How accurately do you think the app recorded the amount of time you spent walking [the total minutes and brisk minutes]?”) about perceived accuracy of the app is included at 6 months (5-point scale, 1=not accurate and 5=very accurate).

#### Qualitative End of Study Interviews

A subsample of intervention participants (approximately n=20-30) will be interviewed after they have completed the 6-month data collection. These telephone or video call interviews will be semistructured (Multimedia Appendix 4 provides the interview guide), audio recorded, and will explore experiences of using the intervention materials, their walking experiences, and their use of the app.

### Data Analysis

The primary endpoint, the difference between trial arms in change from baseline in activPAL-assessed average minutes of brisk walking (≥100 spm) 3 months after randomization, will be analyzed by a statistician blind to intervention allocation on an intention-to-treat basis. Estimates of effect sizes will be presented with 95% CIs and 2-sided 5% *P* values. The statistical analysis plan will be finalized prior to completion of data collection. Cross-sectional analysis will be performed at 3 months (primary endpoint) and 6 months (secondary endpoint) using linear regression to compare the two trial arms, with baseline time spent walking at ≥100 spm included as a covariate. Generalized linear mixed models will also be used to model repeated measurements over time, with individual patient random effects, and intervention, time, and intervention-by-time interaction fixed effects. Other continuous secondary outcomes (ie, average daily total steps, minutes of light PA, standing time and sitting time, GLTEQ, EQ-5D-5L, Functional Assessment of Cancer Therapy-General [FACT-G], Functional Assessment of Chronic Illness Therapy-Fatigue [FACIT-F], Pittsburgh Sleep Quality Index [PSQI], Generalized Anxiety Disorder-7 [GAD-7], Patient Health Questionnaire-9 [PHQ-9], Physical Activity Appraisal Inventory [PAAI], Self-Report Behavioral Automaticity Index [SRBAI], BMI, and waist circumference) will be analyzed using a similar approach. Relationships with baseline participant and disease characteristics will be investigated through covariates for main effects as well as interactions with the trial intervention. Subgroup analyses will be performed by stratification factors used in the randomization procedure and presented using forest plots and *P* values from interaction tests. Exploratory analyses to understand the role of habit strength as a mechanism in the relationship between the trial intervention and PA will be carried out through further regression modeling.

### Cost-Effectiveness Analysis

Cost-effectiveness will be estimated using a health economic model developed for preliminary analysis of the APPROACH pilot feasibility trial [53]. The model is a cohort Markov model with annual cycles and a lifetime horizon, which links PA to mortality from cancer and other causes. The model will be updated using data from the study, reducing key uncertainties identified as part of the preliminary analysis. The model population will be updated using baseline characteristics from the study. PA changes due to the intervention incorporated in the model will be based on weekly METs rather than on the primary trial outcome, as this measurement aligns better with other data already included in the model defining the relationship between PA and mortality [17,103,104]. Additions to the model will be made to incorporate NHS resource use and quality-of-life data gathered as part of the trial, as these were not included in the previous modeling but are likely to have large impacts on cost-effectiveness. Intervention costs will also be updated. The model will estimate costs and quality-adjusted life years using probabilistic sensitivity analysis to enable uncertainty in the results to be captured. Cost-effectiveness will be estimated across the whole population and in relevant subgroups.

### Qualitative Analysis

Qualitative interview data will be analyzed using a framework analysis approach [105]. This approach was chosen because these interviews will inform the process evaluation of the intervention and data will fulfil predetermined information needs about the implementation of the intervention, mechanisms of action, and contextual factors impacting engagement [106]. We also anticipate mapping BCTs against the Behavior Change Taxonomy Ontology [62,63]. The Framework Method involves both deductive and inductive approaches to analyzing the data, where predefined codes are used alongside open coding [107]. There are 7 stages [107]: transcription, familiarization, coding, developing a working analytical framework, applying the analytical framework, charting the data into the framework matrix, and interpreting the data. This approach enables a systematic procedure for reducing the data while remaining flexible enough to identify unexpected themes and perspectives. Indexing of manuscripts will be undertaken by researchers not involved in the delivery of the intervention to minimize reflexivity biases. A portion of transcripts will be independently double-coded (with any ambiguities or differences discussed and resolved). Final reporting of the qualitative interview data collection and analysis process will be reported according to the COREQ (Consolidated Criteria for Reporting Qualitative Research) Checklist [108].

### Data Monitoring

UCL is the trial sponsor. The trial will be overseen by a Trial Management Group consisting of the chief investigators (AF and PL), co-investigators, and research staff employed on the grant. An external Trial Steering Committee, including 2 independent members, all co-investigators, and lay representatives, will be updated regularly on trial progress and will meet as required to provide input. No formal data monitoring committee will be set up, and there are no criteria for stopping the trial as walking is a very low-risk intervention.

### Adverse Event Reporting

All adverse events that the research team become aware of from the point of consent will be assessed by severity (mild, moderate, or severe), causality (not related, unlikely, possibly, probably, definitely, or not assessable), and expectedness (expected or unexpected). In this population, various expected adverse events relating to participant’s cancer diagnosis and treatment may occur (eg, acute illness, symptoms related to treatment), but as these would not be related to the intervention, they will not be recorded. All other adverse events will be recorded, including “mild skin irritation from the adhesive dressing when wearing the activPAL device” as this has been observed previously in older adults [109] and in the pilot [53].

### Ethical Considerations

Favorable ethical opinion for this study was received from the London-Surrey Research Ethics Committee in October 2023 (reference 23/LO/0740). This study describes protocol version 9.0 (dated January 16, 2025), which was approved and implemented in February 2025. All participants will provide written consent.

The trial has been approved by the London-Surrey Research Ethics Committee (reference 23/LO/0740), the Health Research Authority, and the Research and Development department at each recruiting site. Amendments to the protocol will only be implemented after appropriate approval from relevant bodies and communicated where necessary with relevant parties. The study is compliant with the requirements of the General Data Protection Regulation (2016/679) and the UK Data Protection Act (2018). UCL is the data controller, and the study has been registered for Data Protection at UCL (reference Z6364106/2022/07/163 social research). Personal data will only be collected where it is deemed essential to the study. Where possible, personal data are pseudonymized. No identifiable personal data will be used in dissemination of the research. All participants provide informed written consent. Verbally and in the information sheet participants are informed that they are under no obligation to take part and can withdraw at any time without giving a reason and without their medical care being affected. Should any participants ask to withdraw, the data collected up to the point of withdrawal will be analyzed. These participants will also be asked if they allow us to continue to access data from their hospital medical records (collected at 6 months) and linkage with NDRS and HES (if appropriate).

Most study data will be entered directly into, and stored within, the UCL Data Safe Haven secure encrypted platform, but, if on paper, will be stored in locked filing cabinets at UCL. The final dataset will be pseudonymized. All identifiable personal data and research data will be stored for 12 years (allowing time for potential linkage with NHS Digital data 10 years after the trial end date should the research team obtain funding to undertake this) from the trial end date, after which it will be anonymized (removing the study pseudonym and deleting all contact details, including proof of consent). This anonymized dataset (excluding NDRS and HES data) will be entered into the Open Science Framework. The Trial Master File will be archived at UCL, in accordance with the UCL Retentions Schedule, for a minimum of 5 years from the trial end and no longer than 20 years.

## Results

The study was funded in June 2019. Trial recruitment began in November 2023. As of December 2025, a total of 473 participants have been randomized. It is anticipated that the recruitment target will be reached during 2025, and the final participant will complete the study in 2026. The publication of the main results is expected in autumn 2027 after all follow-up data collection and analysis are complete.

## Discussion

### Overview

This study describes the protocol for APPROACH, a multicenter, phase III, randomized control trial, which follows on from the pilot [44,53]. The results will provide robust evidence relating to the clinical and cost-effectiveness of the APPROACH brisk walking intervention among a mixed cohort of individuals diagnosed with breast, colorectal, or prostate cancer, including whether the intervention increases objective, activPal-assessed PA and other outcomes. This study is set within the context of the increasing recognition of the importance of PA in both prehabilitation and rehabilitation settings [6] and growing emphasis on PA advice to be routinely given to individuals diagnosed with cancer [29]. With the introduction of support programs and services being offered to patients locally and nationally [110], the clinical and cost-effectiveness of different types of PA interventions need to be established to support their implementation into routine clinical care.

In addition to the previously outlined small changes to the intervention materials (eg, reformatting the walking planning cards) based on feedback from the pilot [53,111], the following changes were made and incorporated into the original protocol registered in September 2023 (registration number ISRCTN14149329). First, the decision was taken to focus on localized disease. Although systematic reviews have illustrated the safety and potential benefits of PA among metastatic patients [112,113], the limited number of patients recruited to the pilot [53] with metastatic disease (7/90, 7.8%; mainly owing to challenges of obtaining clinical sign-off) meant the feasibility and acceptability of the intervention among this patient group could not be assessed. Second, due to the need to adhere to the research ethics committee requirements, an initial consent-to-contact stage [114] was added. Similarly, to ease the logistics of medical record screening for hospital-based staff, the criteria of time since diagnosis (<2 years) was simplified from the previous pilot criteria (<6 months post end of active treatment). Finally, to give additional flexibility for recruiting hospital sites, a supplementary recruitment route was introduced that allowed the use of leaflets and advertisements (which were reviewed and edits made after following our PPI members’ suggestions) to provide patients with the opportunity to self-refer their interest.

### Strengths

Compared to some more time- and resource-intensive programs, the theory-informed design of APPROACH provides a potentially low-cost intervention, and it is hoped it could be integrated into usual care or existing support services if shown to be clinical and cost-effective. Participants require no specialist equipment (apart from a mobile phone, ownership of which Office for National Statistics figures illustrate is high and increasing in older adults [115]) and are not required to attend any in-person appointments. Therefore, the intervention has the potential to be flexibly accommodated into daily life (eg, around work/life commitments) and does not discriminate against groups who are not able or do not wish to attend time-intensive time-specific PA sessions. This study also objectively measures PA using thigh-worn accelerometers [75]. The researchers involved in organizing study assessments and the statistician undertaking the primary endpoint data analysis will be blinded to group allocation.

### Limitations

This study has several limitations that should be noted. A known limitation of PA research is how study participation (including objective measurement using accelerometers) may influence control group participants to be more active [116]. However, a systematic review by Freene et al [117] suggests these are not significant improvements. Apart from the accelerometer measurement of PA, many of the other outcomes will be self-reported (including use of the app) and therefore have the potential to introduce bias. App engagement will also be explored in the qualitative end-of-study interviews, which, although self-reported, may provide further context on the frequency of use, users’ sense of engagement with the app, and reasons for continued or discontinued use. Furthermore, the eligibility screening question relating to current levels of MVPA (ie, those already meeting 150 minutes of MVPA per week are ineligible) is self-reported, which again may introduce bias [118,119] and result in some active participants being recruited [120]. Research by Smith et al [121] indicates that patients diagnosed with prostate cancer tend to overestimate their PA levels, suggesting we may be conservative in our inclusion. Furthermore, the alternative of obtaining objective measures of PA during the screening phase was not practical for this study.

We acknowledge that the nature of this app-based intervention may not appeal to some patients who do not own a smartphone or consider themselves technologically literate. Office for National Statistics figures illustrate smartphone ownership is increasing in older adults [115], and the APPROACH team provide telephone support to all to increase confidence where needed. Future studies could also fund and provide devices for participants to reduce any digital exclusion or partner with charities set up to support digital poverty when delivering the intervention. As the app being explored in this study is publicly available, there is a possibility that control group participants will find and use it independently. This was not seen in the pilot study [44,53] among 90 participants and is being monitored in the trial follow-up. Furthermore, all participants are asked about their use of apps at baseline (to screen out those already using the study app) and at the follow-ups. Furthermore, the intervention itself is a package of support, and use of the app in isolation (without the broader behavioral support) is not considered equivalent to being in the intervention group. Finally, using a publicly available app meant we were unable to obtain and interrogate app diagnostic data for the trial participants as this option was not available at the time of setting up the trial.

### Dissemination

Study participants will receive a summary of the study findings after data analysis has taken place. Nonidentifiable study results will be disseminated widely in peer-reviewed publications, conference presentations, social media outlets (eg, LinkedIn), and through our PPI representatives. Direct quotations from interview data will be published anonymously.

### Conclusion

To conclude, if found to have a positive effect on PA at 3 months, APPROACH has the potential to provide health professionals with a cost-effective package of support that can be offered to patients diagnosed with cancer. If implemented, this could contribute to improved physical and psychosocial long-term outcomes for those living with and beyond cancer.

## Appendix

Multimedia Appendix 1 SPIRIT 2013 checklist.

Multimedia Appendix 2 Online consent form.

Multimedia Appendix 3 TIDiER checklist - APPROACH randomized controlled trial.

Multimedia Appendix 4 APPROACH randomized controlled trial interview guide.

Multimedia Appendix 5 Peer review report from the Yorkshire Cancer Research Advisory Committee.

## Abbreviations

BCT: behavior change technique
CONSORT: Consolidated Standards of Reporting Trials
COREQ: Consolidated Criteria for Reporting Qualitative Research
CSRI: Client Service Receipt Inventory
FACIT-F: Functional Assessment of Chronic Illness Therapy-Fatigue
FACT-G: Functional Assessment of Cancer Therapy-General
GAD-7: Generalized Anxiety Disorder-7
GLTEQ: Godin Leisure Time Exercise Questionnaire
HES: Hospital Episode Statistics
MET: metabolic equivalent of task
MVPA: moderate-to-vigorous physical activity
NDRS: National Disease Registration Service
NHS: National Health Service
PA: physical activity
PAAI: Physical Activity Appraisal Inventory
PHQ-9: Patient Health Questionnaire-9
PPI: patient and public involvement
PSQI: Pittsburgh Sleep Quality Index
REDCap: Research Electronic Data Capture
SPIRIT: Standard Protocol Items: Recommendations for Interventional Trials
spm: steps per minute
SRBAI: Self-Report Behavioral Automaticity Index
TIDieR: Template for Intervention Description and Replication
UCL: University College London

## Data Availability

Data sharing is not applicable to this study, as no datasets were generated or analyzed during this study. The full intervention materials are not supplied with the protocol paper since recruitment for the study is ongoing, and we do not want the materials to be publicly available until the end of the trial.
