# Supplementary material for: An App-Based Behavioral Support Intervention Promoting Physical Activity (APPROACH) in Patients Diagnosed With Breast, Prostate, or Colorectal Cancer: Protocol for a Randomized Controlled Trial
Source: JMIR Res Protoc. 2026 Jan 13;15:e77096. doi: 10.2196/77096 (PMC12848493; doi:10.2196/77096)
Supplement: Multimedia Appendix 2 [file resprot_v15i1e77096_app2.docx]

**ONLINE CONSENT FORM**

***Study title*: APPROACH – an app for health & wellbeing after cancer – a randomised controlled trial**

If you would like to take part in the APPROACH study please confirm your agreement with the statements below. For statement where there is the option to respond yes or no this aspect of the study is optional and you will still be able to participate if you select No. You need to agree to all the other statements to be able to participate.

| 1. I confirm that I have read and understand the information sheet, version 5.0 27/09/23 for the above study and have had the opportunity to ask questions. | ***Radio button*** |
| --- | --- |
| 1. I understand that my participation in this trial is voluntary and that I am free to withdraw at any time, without giving any reason, and without my medical care or legal rights being affected. | ***Radio button*** |
| 1. I understand that relevant sections of my hospital medical notes may be looked at by individuals from the relevant NHS trusts and approved APPROACH team researchers with appropriate contracts at the hospital. The data collected will include: information about your cancer diagnosis and treatment, any other health conditions diagnosed before or during study, and the number of contacts with hospital during study participation. This data will be shared with University College London secure computer systems, and may be looked at by members of the APPROACH research team (from University College London or University of Leeds). Personally identifiable information will only be accessed by individuals from University College London, University of Leeds, [insert trust name] and regulatory authorities who need access to this information to conduct the study. | ***Radio button*** |
| 1. I understand that information collected about me may be used to support other research in the future, and will be anonymously shared publicly. I will not be identified. | ***Radio button*** |
| 1. I agree to completing online questionnaires, and to wearing an activPAL for 7 days on 3 occasions. | ***Radio button*** |
| 1. I understand that any direct quotations I provide may be included anonymously in research reports, teaching, presentations and publications. | ***Radio button*** |
| 1. I understand that my data will be anonymised 12 years after the trial end date (all personally identifiable data, including consent forms will be deleted). | ***Radio button*** |
| 1. I consent to participate in the APPROACH study. | ***Radio button*** |
| 1. I agree to measuring my height, weight and waist circumference and entering this information into an online questionnaire. | **Radio buttons:**  **Yes and No** |
| 1. I agree to being invited to participate in a semi-structured telephone interview at the end of the study and that if I choose to do this, that the interview will be recorded and then transcribed. This is optional and I do not have to agree to be interviewed. | ***Radio button:***  ***Yes and No*** |
| 1. I give consent for the APPROACH study team to access my NHS number from the hospital from which I was recruited and share my NHS number/other information about me with the national bodies that hold the National Disease Registration Service (NDRS) and Hospital Episode Statistics (HES) (currently NHS Digital) in order to identify me in this registry. I understand that this means that information about my cancer diagnosis, hospital visits, treatment information and my health status will be shared with the APPROACH team for up to 12 years after the study has finished. If I withdraw from the study, I will be asked if I am still willing for the APPROACH team to access information about my health status from NDRS/HES, if I had previously provided permission for this. This is optional and I can participate in the study but select No to this. | **Radio buttons:**  **Yes and No** |

**[If Yes to point 11]**

Please provide your NHS number, if possible *(this can usually be found in a recent clinic letter)*

(note: the reason we ask for this as it makes it easier for us to identify your records in the NDRS/HES registries)

**[All to provide]**

Title:

First name:

Surname:

Telephone number:

Second telephone number (if applicable):

Email address:

Home address including postcode:

Signature:

On the next page you will be shown a PDF of all the information you have read here and your responses. You must tick the box on the next page and click submit to fully send the information. You will also be able to download this for your records if you wish. If you would also like us to email you a copy of this PDF please click here. ***Radio button***

**NEXT**

*Page Break*

*A PDF of the information sheet and their consent responses will be shown on the screen and participants can download or print this.*

*Participants then confirm they are happy with their responses and submit this form.*

*If they clicked the radio button to request an email copy of the PDF this will automatically be emailed to the first email address they entered.*
