# Supplementary material for: An App-Based Behavioral Support Intervention Promoting Physical Activity (APPROACH) in Patients Diagnosed With Breast, Prostate, or Colorectal Cancer: Protocol for a Randomized Controlled Trial
Source: JMIR Res Protoc. 2026 Jan 13;15:e77096. doi: 10.2196/77096 (PMC12848493; doi:10.2196/77096)
Supplement: Multimedia Appendix 3 [file resprot_v15i1e77096_app3.docx]

**Supplementary File 3: TIDiER checklist**

| **Name** | APPROACH: app-based intervention with brief behavioural support to promote physical activity in people affected by cancer |
| --- | --- |
| **Why** | Physical activity has been shown to improve multiple physical and psychosocial outcomes after a cancer diagnosis. Thus, people living with and beyond cancer (LWBC) are recommended by the World Cancer Research Fund to engage in ≥150 minutes of at least moderate intensity physical activity per week. However, many people LWBC do not meet these physical activity guidelines. Many people LWBC report receiving little information about physical activity from healthcare professionals, including oncologists. Healthcare professionals report many barriers to giving physical activity advice, including lack of knowledge of guidelines, not feeling like the right person to give advice, and lack of time and resources. Thus, physical activity interventions are needed that could be rolled out to many people LWBC and that could be delivered by healthcare professionals with low cost and ease. Smartphone apps provide a promising platform for scalable behaviour change interventions. However, there is limited research exploring the potential of smartphone apps in physical activity promotion after cancer. Qualitative interview studies with people LWBC have shown that they feel favourably towards apps that promote walking and feel that it is important that such apps are endorsed by cancer clinical nurse specialists. Interviews with cancer clinical nurse specialists have shown that they also feel positive about the role of walking-based apps for people LWBC. A pilot study has already been undertaken to explore a app-based intervention with brief behavioural support promoting brisk walking in people diagnosed with cancer, and study methods were considered feasible and acceptable.  Therefore, the current proposed study aims to evaluate the efficacy and cost-effectiveness on the difference between trial arms in the changes from baseline in activPAL-assessed average minutes of brisk walking (≥100 steps per minute, spm) after 3 months. Secondary outcomes aim to explore the impact on a variety of other clinical (e.g. brisk walking at 6 months, other activity outcomes, quality of life) and cost-effectiveness outcomes. The RCT will be a multicentre, Phase III, two-armed, individually randomised controlled trial and will be conducted in Yorkshire in the UK. |
| **What (materials)** | The intervention group will be recommended to use Active 10, an app developed by Public Health England for the general public. The Active 10 app enables the setting of goals (e.g. 1 to 3 Active 10s per day), provides feedback on the number of minutes walked briskly per day, gives information about the health and emotional consequences of physical activity, enables the setting of reminders to walk, and provides non-specific rewards if walking targets have been met.  Participants in the intervention group will also be given a leaflet containing information about physical activity and cancer, access to a website with local information about walking groups/ideas, and walking planner cards to support active planning and monitoring of their walking plans. Participants will receive additional behaviour change support from the research team via two telephone/video calls, one occuring at baseline and around four weeks later. The content of these materials are based on Habit Theory and include behaviour change techniques which have shown efficacy in promoting physical activity in inactive adults. These calls are intended to closely replicate conversations that a healthcare professional (e.g. Clinical Nurse Specialist) could have with a patient as part of routine care, should this intervention be implemented in clinical practice. |
| **What (procedure)** | Upon randomisation to the intervention group, intervention participants will be posted a letter of endorsement of physical activity and using the app from their clinical team, and the intervention materials (leaflet, website and walking planners), with an appointment time for a researcher to call and deliver the intervention telephone/video call. The materials will encourage participants to download the Active 10 app in advance of the call with the researcher.  During the first telephone/video call, the researchers will discuss the recommended physical activity guidelines for people LWBC; the associated benefits of meeting these guidelines and of increasing physical activity by any amount; work through the walking planner; help with setting daily walking goals; help with developing a plan/habit for opening the app; and help with downloading the app for participants who have not already done so. After the call, participants will be free to use the Active 10 app, leaflet and walking planners at their own discretion. Participants will receive a second call at 4 weeks to check how they are getting on; to remind them of their goals; and to re-cap any of the information from the first call. |
| **Who provides** | The study researchers, who have extensive research experience in working with people LWBC, will provide participants with behavioural change support in the telephone/video calls. |
| **How** | Upon randomisation to the intervention group, intervention participants will be posted a letter of endorsement of physical activity and using the Active 10 app from their clinical team, and the intervention materials, consisting of the leaflet, website and walking planner cards. Participants will receive a one-to-one telephone/video call at baseline and at four weeks for additional behaviour change support. Participants will access the Active 10 app via their smartphone. |
| **Where** | The intervention will be delivered remotely and participants will access all the intervention elements at a location of their choice. |
| **When and how much** | Frequency of using the Active 10 app, walking planner cards, website and leaflet will be at the discretion of the participant. Participants will receive a telephone/video call at baseline and 4 weeks. |
| **Tailoring** | All participants will receive the same intervention materials: Active 10 app, leaflet, website and walking planner cards. However, participants can choose their own Active 10 goals, which will be set in the baseline telephone/video call. These goals can be adapted at any time and will be discussed during the follow-up telephone/video call if participants are not meeting their goals and want to decrease them, or if they are meeting/exceeding their goals and want to increase them. During the calls, and independently, participants will have the opportunity to make their own plans for when and how they will add brisk walking to their days. |
| **How well** | Fidelity of intervention delivery in the telephone/video calls will be assessed by scoring the recordings of the intervention calls against a checklist of content, including the behaviour change techniques included in the script, to assess how many of these techniques were covered. |
