# Supplementary material for: An App-Based Behavioral Support Intervention Promoting Physical Activity (APPROACH) in Patients Diagnosed With Breast, Prostate, or Colorectal Cancer: Protocol for a Randomized Controlled Trial
Source: JMIR Res Protoc. 2026 Jan 13;15:e77096. doi: 10.2196/77096 (PMC12848493; doi:10.2196/77096)
Supplement: Multimedia Appendix 4 [file resprot_v15i1e77096_app4.docx]

**Supplementary File 4: Interview Guide**

*Before completing the interview check if participants completed the online feedback questions* ***(A41/A42).*** *If they have then have a quick look at their responses as it might help inform this conversation.*

*This guide is to ensure key aspects of participants’ opinions of participating in the trial are covered during the interview.  This is a semi-structured interview guide, and as such, a respondent-sensitive approach should be taken, allowing deviation from question order and raising additional issues if appropriate, depending on the conversation with each individual.*

*Interviewers should be mindful of the sensitive subject of the interview.*

**Introduction**

*Introduce yourself and explain that the purpose of the call is to find out more about how they have found participating in the study. Assure participant confidentiality, and ask participant to be as open and honest as possible in their answers and that there are no right/wrong answers – we’re just interested in their opinions. Ask permission to record the phone call so that we can come back to their answers.*

- You were allocated into the group who received extra support to increase activity/move more. Can you talk me through what you remember about the support you received?
  - Prompts: through the post? Over the phone?
  - Prompts: leaflet (about activity + cancer, walking, the Active 10 app), the walking planner, the phone call, the website and the Active 10 app
  - What did you like about (ask about elements individually)?
  - What did you not like about (as about each element individually)?
  - Which parts did you find useful/helpful/informative? (if any?)
  - Did you learn anything new from these resources? What can you remember about the information about physical activity in relation to cancer?
  - What did you think about the focus on walking?
    - Would you have liked it to focus on other types of activity/exercise?
- Did you download Active 10?
  - If yes (Adapt/ask to elaborate on following Qs, where appropriate):
    - Can you talk me through how you found searching for, downloading/installing the app? Did you use the instructions in the leaflet? Were they clear?
    - Did you use it?
    - How did you find using it?
    - How often did you use it? Did that change over time? Do you still use it?
    - Which features did you use? Can you comment on the different features of the app (e.g. setting the daily 10/20/30 minute goal, using the app to check progress towards that goal, distinguishing between brisk walking/normal pace walking, the graphs to show weekly/monthly walking, push notifications/reminders, badges/rewards) – which aspects did you like/use/find useful (if any?) Did they encourage to be more active?
    - Did you set any goals? Did you monitor your activity using the app?
    - Did you enjoy using it?
    - Did you use the app with anyone else?
    - Did you like using an app to track activity?
  - If no (adapt/ask to elaborate on following questions, where appropriate):
    - Why not?
    - Did the other parts of the intervention encourage you to be more active/walk/try any other apps/look for other resources/ways to help you be more active?
- Do you think the app is appropriate for people who have been diagnosed with cancer/undergoing or after treatment? (Ask to elaborate)
- What do you think about using apps to promote activity (walking or any other type of activity/exercise) for people after a cancer diagnosis? (Or apps in general)
  - Prompts: what do you think are the benefits of using apps for activity?/generally?
  - Prompts: what do you think are the drawbacks/negatives of using apps for activity?/generally
- How do you think you’d have felt if you’d been recommended to this app/given the leaflet/had a similar conversation as the phone call by your nurse, doctor, other healthcare professional etc?
- Have you felt anything has changed since taking part in the study? (Prompt: noticed any physical/psychological changes, changes in thoughts/feelings, wellbeing?)
- Do you have any thoughts/comments about how we could change the support you receive to improve it in the future?

**General**

- Have you spoken to any friends/family about taking part in this study?
  - Prompts: has that had any impact?
  - Has there been any positive or negative impact on you taking part in this study because of speaking to others/or not about it? E.g. has it had any impact on how much activity you’ve done? Whether you’ve been able to stick to being more active? Stick to using the app? Was it important to you/could it have helped?
- Can you tell me how you felt, overall, about being a participant in this research?
  - What were the benefits of taking part?
  - What were the negatives of taking part?
- Do you think you have gained anything from taking part in this trial?
- Would you recommend the app and support you received to other people diagnosed with breast/prostate or bowel cancer?
- Is there anything you think we should have done differently?
- Is there anything else you would like to say about the trial, that we’ve not already spoken about?

**Concluding comments/thanks**

*Thank the participant for their time and for speaking to us about their opinions/experiences of taking part in the study. Emphasise that we really appreciate their time for taking part in the research and that it will help to improve cancer care for other people and inform future research studies.*
