# Supplementary material for: An App-Based Behavioral Support Intervention Promoting Physical Activity (APPROACH) in Patients Diagnosed With Breast, Prostate, or Colorectal Cancer: Protocol for a Randomized Controlled Trial
Source: JMIR Res Protoc. 2026 Jan 13;15:e77096. doi: 10.2196/77096 (PMC12848493; doi:10.2196/77096)
Supplement: Multimedia Appendix 5 [file resprot_v15i1e77096_app5.pdf]

REVIEWER COMMENTS ON YCR AWARD APPLICATION FOR 2018 FUNDING ROUND

"Saving 2000 lives a year in Yorkshire by 2025"

yorkshire cancer research
Saving Yorkshire Lives

Name of Applicant	Project Title
**Dr Abigail Fisher**<br>**Ms Anna Roberts**	APPROACH: Using a mobile application to promote physical activity after cancer
**Funding Decision**	**Comments from Research Advisory Meeting**
**FUNDED**	The Committee welcomed this excellent application for an exercise study from an excellent research team. The Committee particularly liked the clear feasibility criteria that will define if a full trial can go ahead, the long-term follow up plans and the cost effectiveness of the study. There were a few suggestions and comments that may help finesse the proposed study:<br>• Would some form of face-to-face motivation help people to engage with the app? Especially given the primary findings suggest only 25% of people would prefer an app to deliver fitness and lifestyle advice.<br>• Parts of Yorkshire have very poor mobile internet connect – this could affect us of the app.<br>• Contamination of the control could be a major issue if not carefully considered*.<br>*It is noted that this issue has been responded to in the query sheet.

REVIEWER 1 COMMENTS	
**IMPORTANCE**	The proposed work is pragmatic and addresses a clear need in enhancing well-being and overall quality of life in cancer survivors (or people LWBC). The researchers clearly outline the benefit to survivors in the Yorkshire area, as well as potentially to the health care system.<br><br>The proposed work presents a realistic timeline and clear description of benefits for potential participants LWBC. Given the background work to date, the expertise of the team, utilization of an existing app, support of the government agency behind the app, and inclusion of key stakeholders, including cancer survivors and health care professionals, I believe this project is ideally set up for success. The team has proposed the initial 15 months for the pilot work, which is aggressive but completely achievable given use of existing resources and team in place. The full scale RCT will take an additional 2.5 years, and with its pragmatic design, will have substantial impact as they build the evidence towards supporting the app as part of standard cancer care.
**IMPACT**	The proposal is an excellent fit for the funding priority cancer rehabilitation. As noted in the call, exercise has substantial evidence for its role in enhancing the quality of life for

	people LWBC. The proposed project is a very pragmatic RCT, utilizing an existing resource (a physical activity app that includes tracking and behaviour change principles), to improve PA levels in people LWBC. This is a very timely proposal as there is a significant need to begin to address wellness in the cancer journey, and provide support for people LWBC to engage in healthy behaviours, such as PA, to address acute and long term negative side effects associated with cancer and its subsequent treatments.<br><br>Successful completion of the work will impact numerous outcomes in the participants LWBC, including physical, psychosocial and overall quality of life; as well as positively impact the health care system (potential cost savings by having a healthier population of people LWBC). Evidence clearly supports the benefits associated with being more active in cancer survivorship. Thus the successful implementation of the app, even if it doesn't get participants up to guideline levels, at any increase of PA will have some positive impact. The work absolutely fits with the goal of saving 2000 lives a year in Yorkshire by 2025, as there is strong evidence for the link between PA levels and mortality. While exercise and cancer work often focuses on achieving "exercise guideline" levels of activity, even small changes (10 min a day of walking) are positively related to outcomes. Small steps in PA behaviour change are clearly linked to longer term PA adherence and thus the beneficial morbidity and mortality outcomes. Perhaps most impressive with this work is the opportunities for scalability. If the app is deemed acceptable for use, by both people LWBC and the health care system that will help to deliver it, and is associated with beneficial changes in PA levels, this work should provide the system with the knowledge on how it can be successfully implemented on a wider scale. Finally, this work is unique - there is a lack of pragmatic trials that focus on implementation of PA resources to enhance quality of life in people LWBC. Using an existing resource that has been piloted and that is supported for use within this project is ideal, from a cost-savings perspective but also for the timeline of moving the "app to market" and having the population of people LWBC using it in a timely fashion.
**METHODOLOGY**	The pilot work will yield results around the feasibility of the app, as well as secondary data on potential outcomes, at 3 months. While acceptability and feasibility of the app within the RCT can be achieved, 3 months may not present a long enough timeline (in either the pilot or the full RCT in phase 2) to see all of the outcomes as presented (I.e., changes in fitness, quality of life, BMI/waist circumference). A few potential changes may be considered:<br>1-Stratfication if on/off active treatment. Given the impact of treatment (acute) on well-being and ability to be active, would suggest that this may be a critical variable in assessing the feasibility of the Active10 app. For example, if those on treatment are less able to increase PA levels in comparison to those completed treatment, that will provide critical information with regards to when the app should be offered within standard cancer care. In addition, uptake of the app through clinician referral and/or signposting, as well as adherence to using the app, may be different depending on this timing question. I believe this is a central issue that will impact both pilot primary outcome (feasibility) as well as the potential benefits of being more active (without adherence to using the app, no benefits will be seen).<br>2-accelerometer data is excellent, but why not also use data collected within the Active10 app on walking minutes? Unsure why that is not included as an outcome measure, as for long-term implementation in standard care, mailing out accelerometers will not be feasible. Thus having a comparison of what the app collects, vs accelerometer and the GLTEQ would be very useful and informative.<br>3-Would like to see a bit more detail about the actual intervention - will there be

	tracking of the Active10 app usage and then feedback provided to them from the research team (i.e. if they are not using, follow up to see if there are issues?). Will the app simply be provided and then no active intervention - just assessments at 3 months (and then 6 months as well in phase 2)? Building some more data gathering from the app (tracking of minutes? steps?) and how the behaviour change components will be presented (within the app) is critical. This work has the potential to show how a commercially available app can change PA behaviour, but understanding if it's only this app and what components are most valued and impactful on behaviour change (and not just at the end via the n=5 qualitative interviews) should be assessed.<br><br>Salary and non-salary costings seem reasonable.
**ADDITIONAL COMMENTS**	First, I would like to commend the team on the proposed work. The field of cancer and exercise needs more trials like this - that utilise existing resources (technology or otherwise) to support PA participation. This very pragmatic approach will be impactful. We are no longer at the stage where we require as much focus on "is exercise good for cancer survivors?", as we know it is. We need work done to now help the health care system actually support people LWBC to be active. This work has the potential to do so, at minimal "cost" to the system, and with the potential to be highly scalable and impact large numbers of people LWBC.<br>Second, the team has done an outstanding job of building the background work to support this application, and has included a solid team to support integrated knowledge translation. The opportunity to build impact and fine tune delivery of the app to promote PA levels during the trial should not be overlooked (even beyond the pilot phase), and the cost savings to the system long term is an important potential outcome. I encourage the team to consider aggressively pursuing funding for long term tracking of participants (i.e. building a cohort database) to assess the economic and quality of life benefits for the population impacted.

**REVIEWER 2 COMMENTS**	
**IMPORTANCE**	There is a clear need to address the burden associated with cancer survivorship, which this application does. Physical activity represents an ideal intervention in many ways, as it has broad appeal, and unlike other intervention options can be effective for improving physical and mental components of quality of life, whilst also reducing overall risk of morbidity and mortality. Despite survivor's interest in becoming more active, it is a difficult thing to do and to maintain. This trial will test if a publically available intervention in combination with minimal additional advice (which could be feasibly provided by nurses) can be used to support cancer survivors (those with breast, prostate and colon cancer) to become more active.<br><br>The use of a publically available app increases the likelihood that this intervention could be disseminated rather rapidly at the conclusion of the trial. Exactly how the app would be 'signposted' and how effective this will be requires further study, but I agree it makes sense first to see if it works and how much it costs. Of note, the team have been consulting with relevant stakeholders and have the expertise to continue this work if effective.

**IMPACT**	The project aligns closely with Yorkshire Cancer Research's funding priority cancer rehabilitation theme.<br>If effective, the intervention is likely to significant impact cancer outcomes. The study is focused on survivors of prostate, breast and colorectal cancer. There is evidence (though observational at this point) that engaging in physical activity improves changes of survival among people with these cancers. Importantly, the promotion of physical activity is to ensure people not only survive but they survive well.<br><br>The work will be of national significance.<br><br>There have been ~2-3 dozen studies looking at the acceptability efficacy of digital interventions for promoting physical activity among cancer survivors. Acceptability data has been quite good, but only 2-3 large fully powered trials have been conducted. This would be one of the first definitive trials, and would be the first trial using an existing app developed by a government body. It is also original in its use of nurses for recruitment, the intervention approach replicating the anticipated dissemination pathway in practice, and the attempt to link data to health registry data to allow long-term follow-up. This would significantly increase the impact of the study. It was also great to see intervention components focused on habit formation, which will be another novel aspect and is much needed. The impact on health outcomes may be improved if the intervention also focused on resistance-training, or included tailored exercise prescription, however the rationale for the selected app is strong (existing government app with strong privacy settings and consistent with survivor preferences) and there is no reason to believe it will not result in significant health benefits as is. These are things that could be considered at a later stage if the base model is found to be effective.
**METHODOLOGY**	There are clear methodological strengths, including the recruitment approach and the use of objective physical activity measures. These address limitations of previous research.<br><br>One area of concern is the capacity for contamination. If the app is freely downloadable those allocated to the usual care group may download it. This is not necessarily a problem, in that downloading the app without support could arguably still be counted as usual care. However, the participant may not have downloaded it otherwise and it may have an intervention effect - which would have an impact on power to detect differences between the groups. The research team does plan to look at usage data of the app, which will help to tease this out at the pilot stage.<br><br>The study design is otherwise clean and will result in clear results by the end of the funding period.<br><br>I cannot comment on the salary costings, however the non-salary costings seem reasonable.

**REVIEWER 3 COMMENTS**	
**IMPORTANCE**	This study addresses a clear clinical need with clear benefits to the population in question which is likely to have scalable impact in potentially large populations at very low cost. (The Active 10 app is readily available for free).

**IMPACT**	This application is very clear and relevant in addressing Yorkshire Cancer Research's funding priority of cancer rehabilitation.<br>Evidence shows that people diagnosed with breast or bowel cancer who increased their PA level by any amount after diagnosis have a 39% reduced mortality risk compared to those who did not. Therefore, even small increases in PA can substantially prolong survival for people LWBC in Yorkshire. This intervention could be offered to the 11,400 people diagnosed with breast, prostate or colorectal cancer per year in Yorkshire. If 1 in 3 of those were to successfully increase PA using the app, this could lead to approximately 440 fewer deaths from these cancers in Yorkshire per year while also substantially reducing the risk of other long-term conditions (e.g. heart disease, diabetes), improving QoL and side effects. This intervention would also meet Yorkshire Cancer Research's aim to "educate and influence better lifestyle decisions that will improve health, reduce the risk of cancer or support successful recovery from cancer". Obviously, the results of this study can be extrapolated to populations outside of those with Cancer so could have impacts on a much wider scale. Some previous work has been done in this area but high quality large scale randomised controlled trials are needed particularly in this population.
**METHODOLOGY**	The methods are well described and are in line with the Medical Research Council guidance for developing and evaluating complex interventions with PPI input and are structured to deliver clear results in the timescale provided.<br><br>I feel the PPI contribution to the process could be more clearly elucidated. In addition, in the Cancer survivor group after the life transforming event of a cancer diagnosis, people often are very clear about their own goals in terms of participating in such a study. Therefore, the inclusion of "Goal Orientated Outcomes" as part of the secondary outcomes would be appropriate.<br><br>Salary and non-salary costs are justified for this application.

**REVIEWER 4 COMMENTS**	
**IMPORTANCE**	Given that the number of people being diagnosed with cancer is on the rise, efforts to improve QoL and survivorship for cancer patients are much needed. The evidence indicates that physical activity can have physiological and psychological benefits for cancer survivors thus the development of an app to improve PA amongst this cohort addresses a clear need and is worthy of investigation.<br>The application demonstrates a clear path to public /patient benefit which is likely to be achieved within suggested timescales. The work could ultimately benefit the NHS by reducing the burden of caring for patients after cancer.
**IMPACT**	The application is well aligned with Yorkshire Cancer Research's funding priority of Cancer Rehabilitation.<br>Successful completion of the work is likely to positively impact cancer outcomes in Yorkshire in particular for those individuals who readily engage with the app and change their behaviour to increase PA. It is well documented that even marginal gains in PA can have significant impact on survivorship.<br><br>The impacts on a wider scale beyond Yorkshire will depend on the efficacy of the pilot RCT and subsequent full trial but given that the app is free to download and the intervention can be rolled out nationally if successful, the wider impacts could be significant.

	The proposed work is outside my area of expertise but does appear to be innovative and original.
**METHODOLOGY**	Give the substantial preliminary work, the use of a free readily available app, the partnership with PHE and a robust study design, the proposed work is likely to yield clear results in the timescales suggested.<br><br>This application is well conceived and methodologically sound. It would be beneficial to have more detail on the actual intervention itself. It is not clear at present what happens after the study participants download the Active 10 app. Downloading an app does not ensure engagement with or adherence to the intervention. Are there any strategies in place to facilitate this? Will there be any individualised support? Will the patient's motivational readiness to change be assessed in any way? Whilst cancer patients are more likely to be motivated to improve their physical activity levels after cancer treatment, behaviour change is difficult and personal support may be needed.<br><br>Both salary and non-salary costings appear appropriate for the proposed work.

REVIEWER 5 COMMENTS	
**IMPORTANCE**	This application addresses a clear need and there is a clear path to public/patient benefit.
**IMPACT**	This application fits with Yorkshire Cancer Research's funding priorities of cancer rehabilitation and clinical trials.<br>The successful completion of this work will impact cancer outcomes in Yorkshire and the application makes a good case for this. The work is not entirely original (see below). If the intervention is effective then it could improve the lives of cancer patients throughout the UK, as well as in other countries.
**METHODOLOGY**	The applicants state that they have been in discussions about a two tier approach to funding. I'm not part to those conversations so have judged the application only on what has been presented. It would have been useful to see the funding break down for the two different part of the application given what has been agreed with Yorkshire Cancer Research.<br><br>App based interventions are becoming increasingly popular and are generally liked by the public. It's not clear what the particular rationale is for using apps with cancer patients? Cancer patients are no different from other patients/people so is there a particular reason why cancer patients will respond differently to an app than the wider population? The evidence for apps in lifestyle change is small-moderate so it is not clear why this particular app needs to be tested in these particular populations? I accept that patients have 'picked' this app but what is the evidence for the app to date? Has the app been tested in the general population and do Public Health England have any plans to do this? Why can't cancer patients be advised to use the app now since it is already freely available? It is unlikely to do harm. Is it worth considering adapting the app to make it cancer patient specific? What is the likelihood the participants are already using similar apps?<br>The intervention is based on a lot of pilot and preparation work which was good to see.<br>It is not clear why the applicants plan to recruit 60 participants in the feasibility trial?<br>It would be useful to consider lending people smartphones in the feasibility work if they don't have them so that this does not preclude people on a low income from the outset.<br>Given the app is freely available now to the population there needs to be some consideration

	for intervention contamination in the control group - this could be substantial. How can controls be stopped from accessing the app?<br>The costs are high given the relatively straightforward methodology.<br>The application is clearly written and well presented. The team look strong.

REVIEWER 6 COMMENTS	
**IMPORTANCE**	This application addresses a clear need, this research addresses the clear need of how best to support cancer survivors in increasing their physical activity levels, which has much potential to bring tangible health benefits. The team has undertaken a lot of important preliminary work and have used the literature very well to develop a very solid rationale to support this bid.<br><br>There is a clear pathway to public/patient benefit but the timescale seems ambitious. It is difficult to say how likely it is that public/patient benefit will be achieved as this depends on the effectiveness of the intervention in regard to increasing physical activity behaviours amongst cancer survivors - and increasing physical activity levels enough to induce such health benefits.
**IMPACT**	This application is closely aligned to Yorkshire Cancer Research's funding priorities of cancer rehabilitation and clinical trials.<br><br>Potentially, if the intervention can increase physical activity levels sufficiently (and sustainably) for health benefits to be accrued. Both the duration and quality of cancer survival could be positively impacted by interventions with proven ability to increase physical activity behaviours.<br><br>It is difficult to say how far this research could go towards the goal of saving 2000 lives a year by 2025 as this depends on the uptake of such an intervention amongst cancer survivors and the level of physical activity behaviour change that can be achieved using this approach. This being said, current observational evidence (of improved survival) is mainly based on people with early-stage (potentially curative) breast and colon cancer, so predictions have to be based on these cancer sub-populations - not all cancer diagnoses in the region (as presented in the application). Effective physical activity interventions could have impact beyond Yorkshire and could bring health benefits beyond survival to many cancer populations, including significant quality of life benefits and better self-management of treatment side effects. This work is sufficiently original and timely, as it is focused on how to best engage cancer survivor populations with new technologies which have the potential to support health behaviour change.
**METHODOLOGY**	I think the pilot work is vital before committing to a fully-powered RCT. Evidence from systematic reviews (e.g. Greaves C et al. 2011; Bourke L et al. 2014; Groen WG et al. 2018) shows that some level of face-to-face support for physical activity behaviour change is important and I am not confident that such a 'remote-support' approach will yield a physiologically-important increase in physical activity behaviour. Groen WG et al. (2018) published a systematic review earlier this year which showed that distance-based physical activity interventions for cancer survivors are not very effective (effect size change in MVPA = 0.21 and no improvement in weekly step-counts). While the proposed 'remote-support' approach is very pragmatic and is designed to overcome implementation challenges/NHS resource limitations, there is a significant trade-off between pragmatism and effectiveness which could impede intervention effectiveness. For this reason, I think an increased level of personalised support (alongside use of the

	App) may be needed to induce physiologically/psychosocially-important changes in physical activity levels - and this highlights the importance of the pilot/feasibility study.<br><br>A systematic review is cited which reported a 39% improvement in survival amongst breast and colon cancer survivors (not prostate cancer survivors, as stated in the proposal) with any amount of increase in physical activity. These observational data need to be heeded with caution, as the authors highlighted evidence of bias in the colorectal cancer studies and most of the breast cancer studies were in women treated for early-stage (potentially curative) disease. This raises the question of whether more stringent inclusion/exclusion criteria are needed: (i) curative breast and colon cancer patients only?; (ii) more precise detail of what level of current or recent physical activity will be deemed an exclusion criterion - as physically active cancer survivors are more likely to volunteer for this study.<br><br>The application lacks detail on what is considered to be a clinically-important change in physical activity behaviour - and this is needed for RCT sample size calculations. In addition, this should be an important secondary outcome of the feasibility study, i.e. demonstrating clear potential that a clinically-important change in physical activity behaviour is achievable in the majority of intervention participants. There is mention of 7000 steps but how many of those steps should be at MVPA intensity? 3000? Could a clinically-important increment in MVPA be defined and justified on the basis of published evidence?<br><br>As sustainable physical activity behaviour change should be another goal of interventions, follow-up beyond 6 months would be worthwhile.<br><br>Although self-reported body mass and waist circumference may be validated for large population studies, blindly assessed objective measures (via RAs) should be the aspiration for smaller-scale RCTs.<br><br>A 2-month study set-up timescale for additional sites (RCT) is highly unlikely to be feasible and it is unclear how many additional sites will be needed to achieve the recruitment target (yet to be determined accurately) within a time-window of 16 months.<br><br>Future work: many of the longer-term outcomes listed for the future cohort study are likely to be underpowered to show meaningful differences between the groups.<br><br><br>For the pilot study the support requested is justified. It is difficult to predict how many patients or centres will be needed for the fully-powered RCT as a minimum clinically important difference has not been considered for the primary outcome. Also, I was surprised to see no mention of CTU involvement/costs in the fully-powered RCT phase of the research.<br>Non-salary costings are justified.
